# Deep Theory of Functional Connections: A New Method for Estimating the Solutions of Partial Differential Equations

**DOI:** 10.3390/make2010004

**Published:** 2020-03-12

**Authors:** Carl Leake, Daniele Mortari

**Affiliations:** Aerospace Engineering, Texas A&M University, College Station, TX 77843, USA

**Keywords:** deep learning, neural network, theory of functional connections, partial differential equation

## Abstract

This article presents a new methodology called Deep Theory of Functional Connections (TFC) that estimates the solutions of partial differential equations (PDEs) by combining neural networks with the TFC. The TFC is used to transform PDEs into unconstrained optimization problems by analytically embedding the PDE’s constraints into a “constrained expression” containing a free function. In this research, the free function is chosen to be a neural network, which is used to solve the now unconstrained optimization problem. This optimization problem consists of minimizing a loss function that is chosen to be the square of the residuals of the PDE. The neural network is trained in an unsupervised manner to minimize this loss function. This methodology has two major differences when compared with popular methods used to estimate the solutions of PDEs. First, this methodology does not need to discretize the domain into a grid, rather, this methodology can randomly sample points from the domain during the training phase. Second, after training, this methodology produces an accurate analytical approximation of the solution throughout the entire training domain. Because the methodology produces an analytical solution, it is straightforward to obtain the solution at any point within the domain and to perform further manipulation if needed, such as differentiation. In contrast, other popular methods require extra numerical techniques if the estimated solution is desired at points that do not lie on the discretized grid, or if further manipulation to the estimated solution must be performed.

## 1. Introduction

Partial differential equations (PDEs) are a powerful mathematical tool that is used to model physical phenomena, and their solutions are used to simulate, design, and verify the design of a variety of systems. PDEs are used in multiple fields including environmental science, engineering, finance, medical science, and physics, to name a few. Many methods exist to approximate the solutions of PDEs. The most famous of these methods is the finite element method (FEM) [1–3]. FEM has been incredibly successful in approximating the solution to PDEs in a variety of fields including structures, fluids, and acoustics. However, FEM does have some drawbacks.

FEM discretizes the domain into elements. This works well for low-dimensional cases, but the number of elements grows exponentially with the number of dimensions. Therefore, the discretization becomes prohibitive as the number of dimensions increases. Another issue is that FEM solves the PDE at discrete nodes, but if the solution is needed at locations other than these nodes, an interpolation scheme must be used. Moreover, extra numerical techniques are needed to perform further manipulation of the FEM solution.

Reference [4] explored the use of neural networks to solve PDEs, and showed that the use of neural networks avoids these problems. Rather than discretizing the entire domain into a number of elements that grows exponentially with the dimension, neural networks can sample points randomly from the domain. Moreover, once the neural network is trained, it represents an analytical approximation of the PDE. Consequently, no interpolation scheme is needed when estimating the solution at points that did not appear during training, and further analytical manipulation of the solution can be done with ease. Furthermore, Ref. [4] compared the neural network method with FEM on a set of test points that did not appear during training (i.e., points that were not the nodes in the FEM solution), and showed that the solution obtained by the neural network generalized well to points outside of the training data. In fact, the maximum error on the test set of data was never more than the maximum error on the training set of data. In contrast, the FEM had more error on the test set than on the training set. In one case, the test set had approximately three orders of magnitude more error than the training set. In short, Ref. [4] presents strong evidence that neural networks are useful for solving PDEs.

However, what was presented in Ref. [4] can still be improved. In Ref. [4] the boundary constraints are managed by adding extra terms to the loss function. An alternative method is to encapsulate the boundary conditions, by posing the solution in such a way that the boundary conditions must be satisfied, regardless of the values of the training parameters in the neural network. References [5,6] manage boundary constraints in this way when solving PDEs with neural networks by using a method similar to the Coons’ patch [7] to satisfy the boundary constraints exactly.

Exact boundary constraint satisfaction is of interest for a variety of problems, particularly when confidence in the constraint information is high. This is especially important for physics informed problems. Moreover, embedding the boundary conditions in this way means that the neural network needs to sample points from the interior of the domain only, not the domain and the boundary. While the methods presented in [5,6] work well for low-dimensional PDEs with simple boundary constraints, they lack a mechanized framework for generating expressions that embed higher-dimensional or more complex constraints while maintaining a neural network with free-to-choose parameters. For example, the fourth problem in the results section cannot be solved using the solution forms shown in Ref. [5,6]. Luckily, a framework that can embed higher-dimensional or more complex constraints has already been invented: The Theory of Functional Connections (TFC) [8,9]. In Ref. [10], TFC was used to embed constraints into support vector machines, but left embedding constraints into neural networks to future work. This research shows how to embed constraints into neural networks with the TFC, and leverages this technique to numerically estimate the solutions of PDEs. Although the focus of this article is a new technique for numerically estimating the solutions of PDEs, the article’s contribution to the machine learning community is farther reaching, as the ability to embed constraints into neural networks has the potential to improve performance when solving any problem that has constraints, not just differential equations, with neural networks.

TFC is a framework that is able to satisfy many types of boundary conditions while maintaining a function that can be freely chosen. This free function can be chosen, for example, to minimize the residual of a differential equation. TFC has already been used to solve ordinary differential equations with initial value constraints, boundary value constraints, relative constraints, integral constraints, and linear combinations of constraints [8,11–13]. Recently, the framework was extended to *n*-dimensions [9] for constraints on the value and arbitrary order derivative of (*n* – 1)-dimensional manifolds. This means the TFC framework can now generate constrained expressions that satisfy the boundary constraints of multidimensional PDEs [14].

## 2. Theory of Functional Connections

The Theory of Functional Connections (TFC) is a mathematical framework designed to turn constrained problems into unconstrained problems. This is accomplished through the use of constrained expressions, which are functionals that represent the family of all possible functions that satisfy the problem’s constraints. This technique is especially useful when solving PDEs, as it reduces the space of solutions to just those that satisfy the problem’s constraints. TFC has two major steps: (1) Embed the boundary conditions of the problem into the constrained expression; (2) solve the now unconstrained optimization problem. The paragraphs that follow will explain these steps in more detail.

The TFC framework is easiest to understand when explained via an example, like a simple harmonic oscillator. Equation (1) gives an example of a simple harmonic oscillator problem.

(1) $$m\frac{{\text{d}}^{2}y}{\text{d}x_{1}^{2}}+k\;y=0\;\text{subject}\;\text{to}:\;\{\begin{array}{l} y(0)=y_{0}\\ y_{x_{1}}(0)=y_{x_{1}}, \end{array}$$

where the subscript in $y_{x_{1}}$ denotes a derivative of *y* with respect to *x*_1_.

Based on the univariate TFC framework presented in Ref. [8], the constrained expression is represented by the functional,

$$f\left(x_{1},g\left(x_{1}\right)\right)=g\left(x_{1}\right)+\sum_{j=1}^{2}\eta_{j}s_{j}\left(x_{1}\right),$$

where the *s*_*j*_(*x*_1_) are a set of mutually linearly independent functions, called support functions, and *η*_*j*_ are coefficient functions that are computed by imposing the constraints. For this example, let’s choose the support functions to be the first two monomials, *s*_1_(*x*_1_) = 1 and *s*_2_(*x*_1_) = *x*_1_. Hence, the constrained expression becomes,

(2) $$f\left(x_{1},g\left(x_{1}\right)\right)=g\left(x_{1}\right)+\eta_{1}+x_{1}\eta_{2}.$$

The coefficient functions, *η*_1_(*x*_1_) and *η*_2_(*x*_1_), are solved by substituting the constraints into the constrained expression and solving the resultant set of equations. For the simple harmonic oscillator this yields the set of equations given by Equations (3) and (4).

(3) $$y(0)=g(0)+\eta_{1}$$

(4) $$y_{x_{1}}(0)=g_{x_{1}}(0)+\eta_{2}.$$

Solving Equation (3) results in *η*_1_ = *y*(0) − *g*(0), and solving Equation (4) yields $\eta_{2}=y_{x_{1}}(0)-g_{x_{1}}(0)$. Substituting *η*_1_ and *η*_2_ into Equation (3) we obtain,

$$f\left(x_{1},g\left(x_{1}\right)\right)=g\left(x_{1}\right)+y(0)-g(0)+x_{1}\left[y_{x_{1}}(0)-g_{x_{1}}(0)\right]$$

which is an expression satisfying the constraints, no matter what the free function, *g*(*x*_1_), is. In other words, this equation is able to reduce the solution space to just the space of functions satisfying the constraints, because for any function *g*(*x*_1_), the boundary conditions will always be satisfied exactly. Therefore, using constrained expressions transforms differential equations into unconstrained optimization problems.

This unconstrained optimization problem could be cast in the following way. Let the function to be minimized, L, be equal to the square of the residual of the differential equation,

$$L\left(x_{1}\right)={\left[m\frac{{\text{d}}^{2}f\left(x_{1},g\left(x_{1}\right)\right)}{\text{d}x_{1}^{2}}+kf\left(x_{1}\right)\right]}^{2}.$$

This function is to be minimized by varying the function *g*(*x*_1_). One way to do this is to choose *g*(*x*_1_) as a linear combination of a set of basis functions, and calculate the coefficients of the linear combination via least-squares or some other optimization technique. Examples of this methodology using Chebyshev orthogonal polynomials to obtain least-squares solutions of linear and nonlinear ordinary differential equations (ODEs) can be found in Refs. [11,12], respectively.

### 2.1. n-Dimensional Constrained Expressions

The previous example derived the constrained expression by creating and solving a series of simultaneous algebraic equations. This technique works well for constrained expressions in one dimension; however, it can become needlessly complicated when deriving these expression in *n* dimensions for constraints on the value and arbitrary order derivative of *n* – 1 dimensional manifolds [9]. Fortunately, a different, more mechanized formalism exists that is useful for this case. The constrained expression presented earlier consists of two parts; the first part is a function that satisfies the boundary constraints, and the second part projects the free-function, *g*(*x*_1_), onto the hyper-surface of functions that are equal to zero at the boundaries. Rearranging Equation (2) highlights these two parts,

$$f\left(x_{1},g\left(x_{1}\right)\right)=\underset{A\left(x_{1}\right)}{\underset{︸}{y(0)+x_{1}y_{x_{1}}(0)}}+\underset{B\left(x_{1},g\left(x_{1}\right)\right)}{\underset{︸}{g\left(x_{1}\right)-g(0)-x_{1}g_{x_{1}}(0)}},$$

where *A*(*x*_1_) satisfies the boundary constraints and *B*(*x*_1_, *g*(*x*_1_)) is a functional projecting the free-function onto the hyper-surface of functions that are equal to zero at the boundaries.

The multivariate extension of this form for problems with boundary and derivative constraints in *n*-dimensions can be written compactly using Equation (5).

(5) $$f(x,g(x))=\underset{A(x)}{\underset{︸}{M_{i_{1},i_{2},\cdots ,i_{n}}(c(x))v_{i_{1}}\left(x_{1}\right)v_{i_{2}}\left(x_{2}\right)\cdots v_{i_{n}}\left(x_{n}\right)}}+\;\;+\underset{B(x,g(x))}{\underset{︸}{g(x)-M_{i_{1},i_{2},\cdots ,i_{n}}(g(x))v_{v_{1}}\left(x_{1}\right)v_{i_{2}}\left(x_{2}\right)\cdots v_{i_{n}}\left(x_{n}\right)}}$$

where $x={\left\{x_{1},x_{2},\cdots ,x_{n}\right\}}^{\text{T}}$ is a vector of the *n* independent variables, M is an *n*-th order tensor containing the boundary conditions *c*(**x**), the $v_{v_{1}},\cdots ,v_{i_{n}}$ are vectors whose elements are functions of the independent variables, *g*(**x**) is the free-function that can be chosen to be any function that is defined at the constraints, and *f*(**x**, *g*(**x**)) is the constrained expression. The first term, *A*(**x**), analytically satisfies the boundary conditions, and the term, *B*(**x**, *g*(**x**)), projects the free-function, *g*(**x**), onto the space of functions that vanish at the constraints. A mathematical proof that this form of the constrained expression satisfies the boundary constraints is given in Ref. [9]. The remainder of this section discusses how to construct the *n*-th order tensor M and the **v** vectors shown in Equation (5).

Before discussing how to build the M tensor and **v** vectors, let’s introduce some mathematical notation. Let *k* ∈ [1, *n*] be an index used to denote the *k*-th dimension. Let ${}^{k}c_{p}^{d}:={\frac{\partial^{d}c(x)}{\partial x_{k}^{d}}|}_{x_{k}=p}$ be *x*_*k*_ = *p* the constraint specified by taking the *d*-th derivative of the constraint function, *c*(**x**), evaluated at the *x*_*k*_ = *p* hyperplane. Further, let ${}^{k}c_{p_{k}}^{d_{k}}$ be the vector of ℓ_*k*_ constraints defined at the *x*_*k*_ = **p**_*k*_ hyperplanes with derivative orders of **d**_*k*_, where **p**_*k*_ and $d_{k}\in {\mathbb{R}}^{\ell_{k}}$. In addition, let’s define a boundary condition operator ${}^{k}b_{p}^{d}$ that takes the *d*-th derivative with respect to *x*_*k*_ of a function, and then evaluates that function at the *x*_*k*_ = *p* hyperplane. Mathematically,

$${}^{k}b_{p}^{d}[f(x)]={\frac{\partial^{d}f(x)}{\partial x_{k}^{d}}|}_{x_{k}=p}.$$

This mathematical notation will be used to introduce a step-by-step method for building the M tensor. This step-by-step process will be be illustrated via a 3-dimensional example that has Dirichlet boundary conditions in *x*_1_ and initial conditions in *x*_2_ and *x*_3_ on the domain *x*_1_, *x*_2_, *x*_3_ ∈ [0, 1] × [0, 1] × [0, 1]. The M tensor is constructed using the following three rules.

1. The element $M_{111}=0$.
2. The first order sub-tensor of M specified by keeping one dimension’s index free and setting all other dimension’s indices to 1 consists of the value 0 and the boundary conditions for that dimension. Mathematically,
  $$M_{1,\ldots ,1,i_{k},1,\ldots ,1}=\left\{0{,}^{k}c_{p_{k}}^{d_{k}}\right\}.$$
  Using the example boundary conditions,
  (6) $$M_{i_{1}11}={\left[0,c\left(0,x_{2},x_{3}\right),c\left(1,x_{2},x_{3}\right)\right]}^{T}\;M_{1i_{2}1}={\left[0,c\left(x_{1},0,x_{3}\right),c_{x_{2}}\left(x_{1},0,x_{3}\right)\right]}^{T}\;M_{11i_{3}}={\left[0,c\left(x_{1},x_{2},0\right),c_{x_{3}}\left(x_{1},x_{2},0\right)\right]}^{T}.$$
3. The remaining elements of the M tensor are those with at least two indices different than one. These elements are the geometric intersection of the boundary condition elements of the first order tensors given in Equation (6), plus a sign (+ or −) that is determined by the number of elements being intersected. Mathematically this can be written as,
  $$M_{i_{1}i_{2}\ldots i_{n}}{=}^{\;1}b_{p_{i_{1}-1}^{1}}^{d_{i_{1}-1}^{1}}\left[{}^{2}b_{p_{i_{2}-1}^{2}}^{d_{i_{2}-1}^{2}}\left[\ldots \left[{}^{n}b_{p_{i_{n}-1}^{n}}^{d_{i_{n}-1}^{n}}[c(x)\right]\ldots \right]\right]{\left(-1\right)}^{m+1},$$
  where *m* is the number of indices different than one. Using the example boundary conditions we give three examples:
  $$M_{133}=-c_{x_{2}x_{3}}\left(x_{1},0,0\right)\;M_{221}=-c\left(0,0,x_{3}\right)\;M_{332}=-c_{x_{2}}\left(1,0,0\right)$$

A simple procedure also exists for constructing the $v_{i_{k}}$ vectors. The $v_{i_{k}}$ vectors have a standard form:

$$v_{i_{k}}={\left\{1,\sum_{i=1}^{\ell_{k}}\alpha_{i1}h_{i}\left(x_{k}\right),\sum_{i=1}^{\ell_{k}}\alpha_{i2}h_{i}\left(x_{k}\right),\ldots ,\sum_{i=1}^{\ell_{k}}\alpha_{i\ell_{k}}h_{i}\left(x_{k}\right)\right\}}^{\text{T}},$$

where *h*_*i*_(*x*_*k*_) are ℓ_*k*_ linearly independent functions. The simplest set of linearly independent functions, and those most often used in the TFC constrained expressions, are monomials, $h_{i}\left(x_{k}\right)=x_{k}^{i-1}$. The ℓ_*k*_ × ℓ_*k*_ coefficients, *α*_*ij*_, can be computed by matrix inversion,

$$\left[\begin{array}{cccc} {}^{k}b_{p_{1}}^{d_{1}}h_{1} & {}^{k}b_{p_{1}}^{d_{1}}\left[h_{2}\right] & \ldots & {}^{k}b_{p_{1}}^{d_{1}}\left[h_{\ell_{k}}\right]\\ {}^{k}b_{p_{2}}^{d_{2}}\left[h_{1}\right] & {}^{k}b_{p_{2}}^{d_{2}}\left[h_{2}\right] & \ldots & {}^{k}b_{p_{2}}^{d_{2}}\left[h_{\ell_{k}}\right]\\ \vdots & \vdots & \ddots & \vdots \\ {}^{k}b_{p_{\ell_{k}}}^{d_{\ell_{k}}}\left[h_{1}\right] & {}^{k}b_{p_{\ell_{k}}}^{d_{\ell_{k}}}\left[h_{2}\right] & \ldots & {}^{k}b_{p_{\ell_{k}}}^{d_{\ell_{k}}}\left[h_{\ell_{k}}\right] \end{array}\right]\left[\begin{array}{cccc} \alpha_{11} & \alpha_{12} & \ldots & \alpha_{1\ell_{k}}\\ \alpha_{21} & \alpha_{22} & \ldots & \alpha_{2\ell_{k}}\\ \vdots & \vdots & \ddots & \vdots \\ \alpha_{\ell_{k}1} & \alpha_{\ell_{k}2} & \ldots & \alpha_{\ell_{k}\ell_{k}} \end{array}\right]=\left[\begin{array}{cccc} 1 & 0 & \ldots & 0\\ 0 & 1 & \ldots & 0\\ \vdots & \vdots & \ddots & \vdots \\ 0 & 0 & \ldots & 1 \end{array}\right].$$

Using the example boundary conditions, let’s derive the $v_{i_{1}}$ vector using the linearly independent functions *h*_1_ = 1 and *h*_2_ = *x*_1_.

$$\left[\begin{array}{ll} 1 & 0\\ 1 & 1 \end{array}\right]\left[\begin{array}{ll} \alpha_{11} & \alpha_{12}\\ \alpha_{21} & \alpha_{22} \end{array}\right]=\left[\begin{array}{ll} 1 & 0\\ 0 & 1 \end{array}\right]\;\to \;\left[\begin{array}{ll} \alpha_{11} & \alpha_{12}\\ \alpha_{21} & \alpha_{22} \end{array}\right]=\left[\begin{array}{cc} 1 & 0\\ -1 & 1 \end{array}\right]\;\;v_{i_{1}}={\left\{1,1-x_{1},x_{1}\right\}}^{\text{T}}.$$

For more examples and a mathematical proof that these procedures for generating the M tensor and the **v** vectors form a valid constrained expression see Ref. [9].

### 2.2. Two-Dimensional Example

This subsection will give an in depth example for a two-dimensional TFC case. The example is originally from problem 5 of Ref. [5], and is one of the PDE problems analyzed in this article. The problem is shown in Equation (7).

(7) $$\nabla^{2}z(x,y)=e^{-x}\left(x-2+y^{3}+6y\right)\text{subject}\;\text{to:}\;\left\{ \begin{array}{l} z(x,0)=c(x,0)=xe^{-x}\\ z(0,y)=c(0,y)=y^{3}\\ z(x,1)=c(x,1)=e^{-x}(x+1)\\ z(1,y)=c(1,y)=\left(1+y^{3}\right)e^{-1} \end{array}\right.\;\text{where}\;(x,y)\in [0,1]\times [0,1]$$

Following the step-by-step procedure given in the previous section we will construct the M tensor:

1. The first element is $M_{111}=0$.
2. The first order sub-tensors of M are:
  $$M_{i_{1}1}=\left\{\begin{array}{lll} 0 & c(0,y) & c(1,y) \end{array}\right\}\;M_{1i_{2}}=\left\{\begin{array}{lll} 0 & c(x,0) & c(x,1) \end{array}\right\}$$
3. The remaining elements of M are the geometric intersection of elements from the first order sub-tensors.
  $$M_{22}=-c(0,0)\;M_{23}=-c(1,0)\;M_{32}=-c(0,1)\;M_{33}=-c(1,1)$$
  Hence, the M tensor is,
  $$M_{i_{1}i_{2}}=\left[\begin{array}{ccc} 0 & c(0,y) & c(1,y)\\ c(x,0) & -c(0,0) & -c(1,0)\\ c(x,1) & -c(0,1) & -c(1,1) \end{array}\right]=\left[\begin{array}{ccc} 0 & y^{3} & \left(1+y^{3}\right)e^{-1}\\ xe^{-x} & 0 & -e^{-1}\\ e^{-x}(x+1) & -1 & -2e^{-1} \end{array}\right]$$

Following the step-by-step procedure given in the previous section we will construct the **v** vectors. For $v_{i_{1}}$, let’s choose the linearly independent functions *h*_1_ = 1 and *h*_2_ = *x*.

$$\left[\begin{array}{ll} 1 & 0\\ 1 & 1 \end{array}\right]\left[\begin{array}{ll} \alpha_{11} & \alpha_{12}\\ \alpha_{21} & \alpha_{22} \end{array}\right]=\left[\begin{array}{ll} 1 & 0\\ 0 & 1 \end{array}\right]\;\to \;\left[\begin{array}{ll} \alpha_{11} & \alpha_{12}\\ \alpha_{21} & \alpha_{22} \end{array}\right]=\left[\begin{array}{cc} 1 & 0\\ -1 & 1 \end{array}\right]\;\;v_{i_{1}}={\left\{1,1-x,x\right\}}^{\text{T}}.$$

For $v_{i_{2}}$ let’s choose the linearly indpendent functions *h*_1_ = 1 and *h*_2_ = *y*.

$$\left[\begin{array}{ll} 1 & 0\\ 1 & 1 \end{array}\right]\left[\begin{array}{ll} \alpha_{11} & \alpha_{12}\\ \alpha_{21} & \alpha_{22} \end{array}\right]=\left[\begin{array}{ll} 1 & 0\\ 0 & 1 \end{array}\right]\;\to \;\left[\begin{array}{ll} \alpha_{11} & \alpha_{12}\\ \alpha_{21} & \alpha_{22} \end{array}\right]=\left[\begin{array}{cc} 1 & 0\\ -1 & 1 \end{array}\right]\;\;v_{i_{2}}={\left\{1,1-y,y\right\}}^{\text{T}}.$$

Now, we use the constrained expression form given in Equation (5) to finish building the constrained expression.

(8) $$f(x,y,g(x,y))=g(x,y)+\frac{xy\left(y^{2}-1\right)}{e}+e^{-x}(x+y)+\;\;+(1-x)\left(g(0,0)+y\left(g(0,1)+y^{2}-g(0,0)-1\right)\right)+(x-1)g(0,y)+\;\;+x(yg(1,1)+(1-y)g(1,0))-xg(1,y)+(y-1)g(x,0)-yg(x,1)$$

Notice, that Equation (8) will always satisfy the boundary conditions of the problem regardless of the value of *g*(*x*, *y*). Thus, the problem has been transformed into an unconstrained optimization problem where the cost function, L, is the square of the residual of the PDE,

$$L(x,y,g(x,y))={\left(\nabla^{2}f(x,y,g(x,y))-e^{-x}\left(x-2+y^{3}+6y\right)\right)}^{2}.$$

For ODEs, the minimization of the cost function was accomplished by choosing *g* to be a linear combination of orthogonal polynomials with unknown coefficients, and performing least-squares or some other optimization technique to find the unknown coefficients. For two dimensions, one could make *g*(*x*, *y*) the product of two linear combinations of these orthogonal polynomials, calculate all of the cross-terms, and then solve for the coefficients that multiply all terms and cross-terms using least-squares or non-linear least-squares. However, this will become computationally prohibitive as the dimension increases. Even at two dimensions, the number of basis functions needed, and thus the size of the matrix to invert in the least-squares, becomes large. An alternative solution, and the one explored in this article, is to make the free function, *g*(*x*, *y*), a neural network.

## 3. PDE Solution Methodology

Similar to the last section, the easiest way to describe the methodology is with an example. The example used throughout this section will be the PDE given in Equation (7).

As mentioned previously, Deep TFC approximates solutions to PDEs by finding the constrained expression for the PDE and choosing a neural network as the free function. For all of the problems analyzed in this article, a simple, fully connected neural network was used. Each layer of a fully connected neural network consists of non-linear activation functions composed with affine transformations of the form A=W⋅x+b, where *W* is a matrix of the neuron weights, **b** is a vector of the neuron biases, and **x** is a vector of inputs from the previous layer (or the inputs to the neural network if it is the first layer). Then, each layer is composed to form the entire network. For the fully connected neural networks used in this paper, the last layer is simply a linear output layer. For example, a neural network with three hidden layers that each use the non-linear activation function *ϕ* can be written mathematically as,

$$N(x;\theta )=W_{4}\cdot \phi \left(W_{3}\cdot \phi \left(W_{2}\cdot \phi \left(W_{1}\cdot x+b_{1}\right)+b_{2}\right)+b_{3}\right)+b_{4},$$

where N is the symbol used for the neural network, **x** is the vector of inputs, *W*_*k*_ are the weight matrices, **b**_*k*_ are the bias vectors, and *θ* is a symbol that represents all trainable parameters of the neural network; the weights and biases of each layer constitute the trainable parameters. Note that the notation N(x,y,…;θ) is also used in this paper for independent variables *x*, *y*, … and trainable parameters *θ*.

Thus, the constrained expression, given originally in Equation (8), now has the form given in Equation (9).

(9) $$f(x,y;\theta )=N(x,y;\theta )+\frac{xy\left(y^{2}-1\right)}{e}+e^{-x}(x+y)+\;\;+(1-x)\left(N(0,0;\theta )+y\left(N(0,1;\theta )+y^{2}-N(0,0;\theta )-1\right)\right)+(x-1)N(0,y;\theta )+\;\;+x(yN(1,1;\theta )+(1-y)N(1,0;\theta ))-xN(1,y;\theta )+(y-1)N(x,0;\theta )-yN(x,1;\theta )$$

In order to estimate the solution to the PDE, the parameters of the neural network have to be optimized to minimize the loss function, which is taken to be the square of the residual of the PDE. For this example,

$$L=\sum_{i}^{N}L_{i}\left(x_{i},y_{i};\theta \right)\;\mathrm{where}\;L_{i}\left(x_{i},y_{i};\theta \right)={\left(\nabla^{2}f\left(x_{i},y_{i};\theta \right)-e^{-x_{i}}\left(x_{i}-2+y_{i}^{3}+6y_{i}\right)\right)}^{2}.$$

The attentive reader will notice that training the neural network will require, for this example, taking two second order partial derivatives of *f* (*x*, *y*; *θ*) to calculate $L_{i}$, and then taking gradients of L with respect to the neural network parameters, *θ*, in order to train the neural network.

To take these higher order derivatives, TensorFlow’s™ gradients function was used [15]. This function uses automatic differentiation [16] to compute these derivatives. However, one must be conscientious when using the gradients function to ensure they get the desired gradients.

When taking the gradient of a vector, *y*_*j*_, with respect to another vector, *x*_*i*_, TensorFlow™computes,

$$z_{i}=\frac{\partial }{\partial x_{i}}\left(\sum_{j=1}^{N}y_{j}\right)$$

where *z*_*i*_ is a vector of the same size as *x*_*i*_. The only example where it is not immediately obvious that this gradient function will give the desired gradient is when computing ∇^2^ *f*_*i*_. The desired output of this calculation is the following vector,

$$z_{i}={\left\{\frac{\partial^{2}f_{1}}{\partial x_{1}^{2}}+\frac{\partial^{2}f_{1}}{\partial y_{1}^{2}},\cdots ,\frac{\partial^{2}f_{N}}{\partial x_{N}^{2}}+\frac{\partial^{2}f_{N}}{\partial y_{N}^{2}}\right\}}^{T},$$

where *z*_*i*_ has the same size as *f*_*i*_ and (*x*_*i*_, *y*_*i*_) is the point used to generate *f*_*i*_. TensorFlow’s™ gradients function will compute the following vector,

$${\tilde{z}}_{i}={\left\{\frac{\partial^{2}\left(\sum_{j=1}^{N}f_{j}\right)}{\partial x_{1}^{2}}+\frac{\partial^{2}\left(\sum_{j=1}^{N}f_{j}\right)}{\partial y_{1}^{2}},\cdots ,\frac{\partial^{2}\left(\sum_{j=1}^{N}f_{j}\right)}{\partial x_{N}^{2}}+\frac{\partial^{2}\left(\sum_{j=1}^{N}f_{j}\right)}{\partial y_{N}^{2}}\right\}}^{\text{T}}.$$

However, because *f*_*i*_ only depends on the point (*x*_*i*_, *y*_*i*_) and the derivative operator commutes with the sum operator, TensorFlow’s™ gradients function will compute the desired vector (i.e., ${\tilde{z}}_{i}=z_{i}$). Moreover, the size of the output vector will be correct, because the input vectors, *x*_*i*_ and *y*_*i*_, have the same size as *f*_*i*_.

### Training the Neural Network

Three methods were tested when optimizing the parameters of the neural networks:

1. Adam optimizer [17]: A variant of stochastic gradient descent (SGD) that combines the advantages of two other popular SGD variants: AdaGrad [18] and RMSProp [19].
2. Broyden–Fletcher–Goldfarb–Shanno [20] (BFGS): A quasi-Newton method designed for solving unconstrained, non-linear optimization problems. This method was chosen based on its performance when optimizing neural network parameters to estimate PDE solutions in Ref. [5].
3. Hybrid method: Combines the first two methods by applying them in series.

For all four problems shown in this article, the solution error when using the BFGS optimizer was lower than with the other two methods. Thus, in the following section, the results shown use the BFGS optimizer.

The BFGS optimizer is a local optimizer, and the weights and biases of the neural networks are initialized randomly. Therefore, the solution error when numerically estimating PDEs will be different each time. However, Deep TFC guarantees that the boundary conditions are satisfied, and the loss function is the square of the residual of the PDE. Therefore, the loss function indicates how well Deep TFC is estimating the solution of the PDE at the training points. Moreover, because Deep TFC produces an analytical approximation of the solution, the loss function can be calculated at any point. Therefore, after training, one can calculate the loss function at a set of test points to determine whether the approximate solution generalizes well or has over fit the training points.

Due to the inherit stochasticity of the method, each Deep TFC solution presented in the results section that follows is the solution with the lowest mean absolute error of 10 trials. In other words, for each problem, the Deep TFC methodology was performed 10 times, and the best solution of those 10 trials is presented. Moreover, to show the variability in the Deep TFC method, problem 1 contains a histogram of the maximum solution error on a test set for 100 Monte Carlo trials.

## 4. Results

This section compares the estimated solution found using Deep TFC with with the analytical solution. Four PDE problems are analyzed. The first is the example PDE given in Equation (7), and the second is the wave equation. The third and fourth PDEs are simple solutions to the incompressible Navier–Stokes equations.

### 4.1. Problem 1

The first problem analyzed was the PDE given by Equation (7), copied below for the reader’s convenience.

$$\nabla^{2}z(x,y)=e^{-x}\left(x-2+y^{3}+6y\right)\;\text{subject}\;\text{to:}\;\left\{ \begin{array}{l} z(x,0)=xe^{-x}\\ z(0,y)=y^{3}\\ z(x,1)=e^{-x}(x+1)\\ z(1,y)=\left(1+y^{3}\right)e^{-1} \end{array}\right.\;\text{where}\;(x,y)\in [0,1]\times [0,1]$$

The known analytical solution for this problem is,

$$z=e^{-x}\left(x+y^{3}\right).$$

The neural network used to estimate the solution to this PDE was a fully connected neural network with 6 hidden layers, 15 neurons per layer, and 1 linear output layer. The non-linear activation function used in the hidden layers was the hyperbolic tangent. Other fully connected neural networks with various sizes and non-linear activation functions were tested, but this combination of size and activation function performed the best in terms of solution error. The biases of the neural network were all initialized as zero, and the weights were initialized using TensorFlow’s™ implementation of the Xavier initialization with uniform random initialization [21]. One hundred training points, (*x*, *y*), evenly distributed throughout the domain were used to train the neural network.

**Figure 1.**
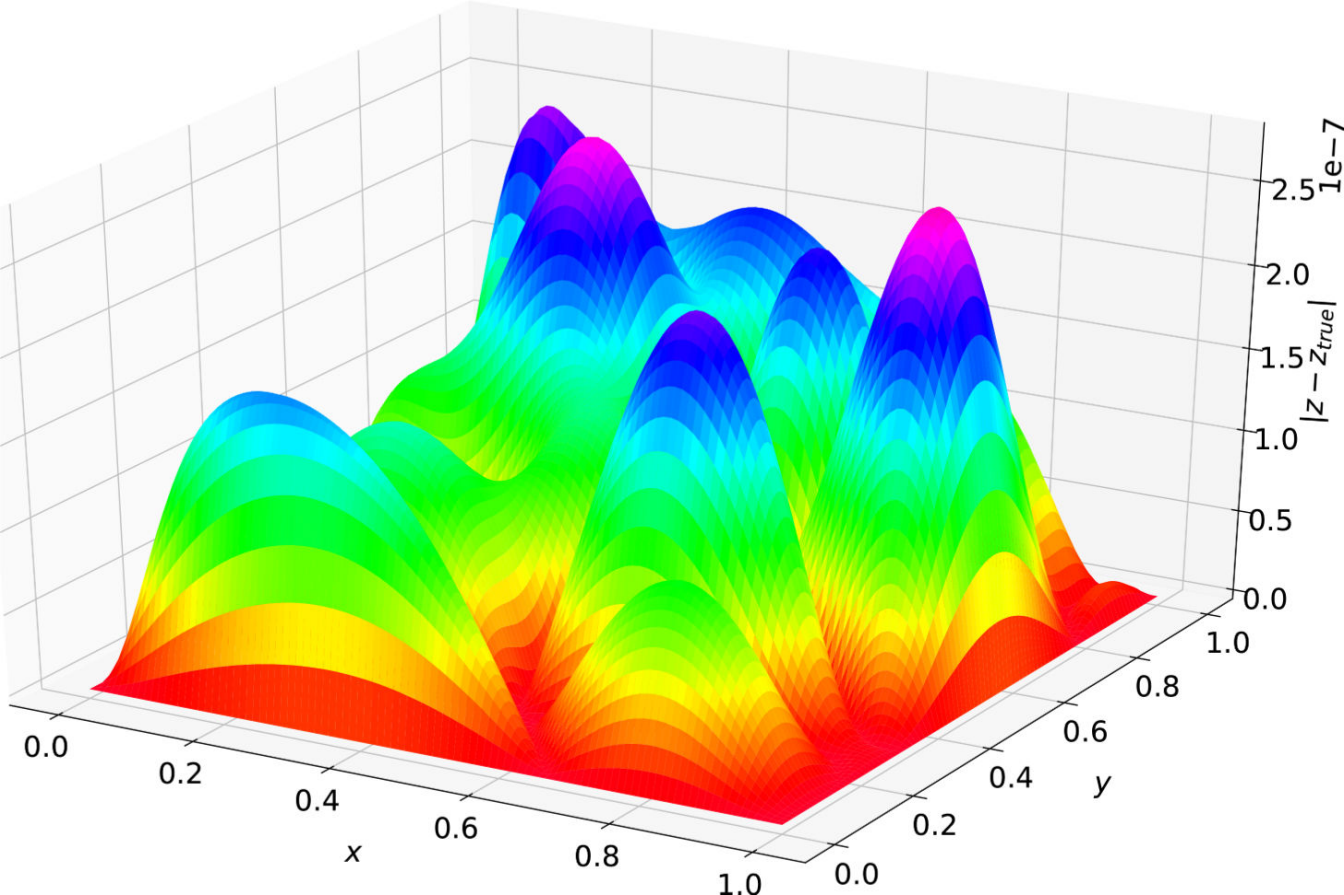
Problem 1 solution error.

**Figure 2.**
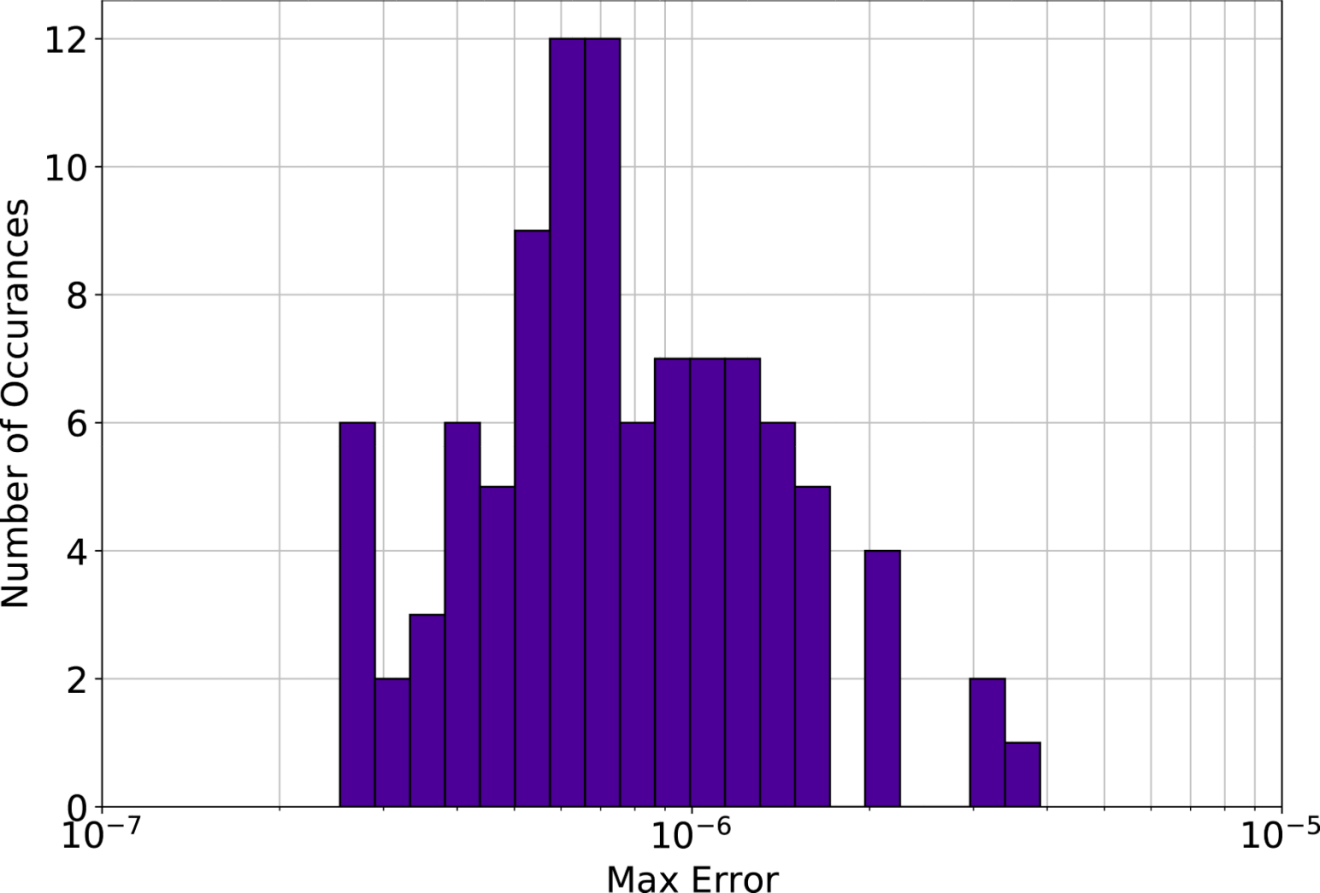
Problem 1 maximum test set solution error from 100 Monte Carlo trials.

Figure 1 shows the difference between the analytical solution and the estimated solution using Deep TFC on a grid of 10,000 evenly distributed points. This grid represents the test set. Figure 2 shows a histogram of the maximum solution error on the test set for 100 Monte Carlo trials.

The maximum error on the test set shown in Figure 1 was 2.780 × 10^−7^ and the average error was 8.517 × 10^−8^. Figure 2 shows that Deep TFC produces a solution at least as accurate as the solution in Figure 1 approximately 10% of the time. The remaining 90% of the time the solution error will be larger. Moreover, Figure 2 shows that the Deep TFC method is consistent. The maximum solution error in the 100 Monte Carlo tests was 3.891 × 10^−6^, approximately an order of magnitude larger than the maximum solution error shown in Figure 1. The maximum error from Figure 1 is relatively low, six orders of magnitude lower than the solution values, which are on the order of 10^−1^. Table 1 compares the maximum error on the test and training sets obtained with Deep TFC with the method used in Ref. [5] and FEM. Note, the FEM data was obtained from Table 1 of Ref. [5].

Table 1 shows that Deep TFC is slightly more accurate than the method from Ref. [5]. Moreover, in consonance with the findings from Ref. [5], the FEM solution performs better on the training set than Deep TFC, but worse on the solution set. Note also “that the accuracy of the finite element method decreases as the grid becomes coarser, and that the neural approach considers a mesh of 10 × 10 points while in the finite element case a 18 × 18 mesh was employed” [5].

The neural network used in this article is more complicated than the network used in Ref. [5], even though the two solution methods produce similarly accurate solutions. The constrained expression, *f* (*x*, *y*; *θ*), created using TFC, which is used as the assumed solution form, is more complex both in the number of terms and the number of times the neural network appears than the assumed solution form in Ref. [5]. For the reader’s reference, the assumed solution form for problem 1 from Ref. [5] is shown in Equation (10). Equation (10) was copied from Ref. [5], but the notation used has been transformed to match that of this paper; furthermore, a typo in the assumed solution form from Ref. [5] has been corrected here.

(10) $$f(x,y;\theta )=x(1-x)y(1-y)N(x,y;\theta )+(1-x)y^{3}+x\left(1+y^{3}\right)e^{-1}\;\;+(1-y)x\left(e^{-x}-e^{-1}\right)+y\left((1+x)e^{-x}-\left(1-x+2e^{-1}\right)\right)$$

To investigate how the assumed solution form affects the accuracy of the estimated solution, a comparison was made between the solution form from Ref. [5] and the solution form created using TFC in this article, while keeping all other variables constant. Furthermore, in this comparison, the neural network architecture used is identical to the neural network architecture given in Ref. [5] for this problem: one hidden layer with 10 neurons that uses a sigmoid non-linear activation function and a linear output layer. Each network was trained using the BFGS optimizer. The training points used were 100 evenly distributed points throughout the domain.

**Figure 3.**
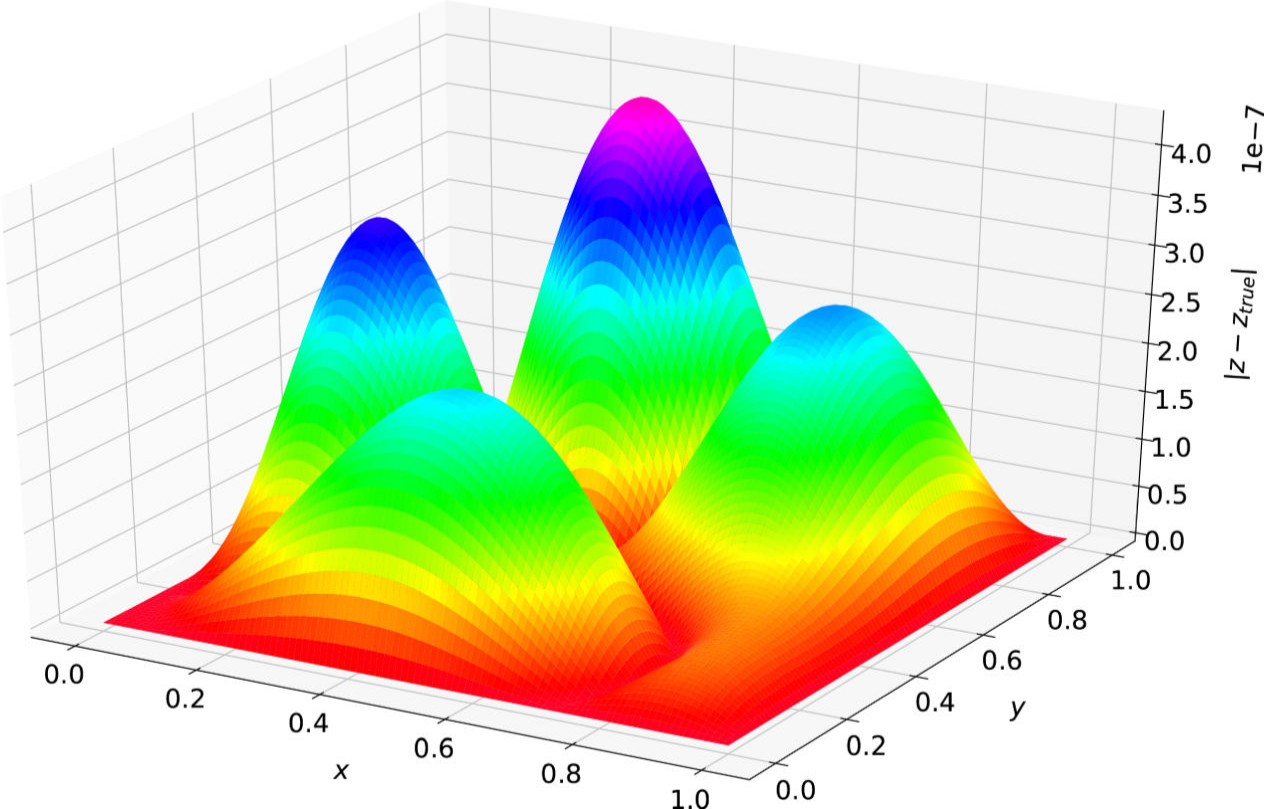
Problem 1 solution error using Ref. [5] solution form.

**Figure 4.**
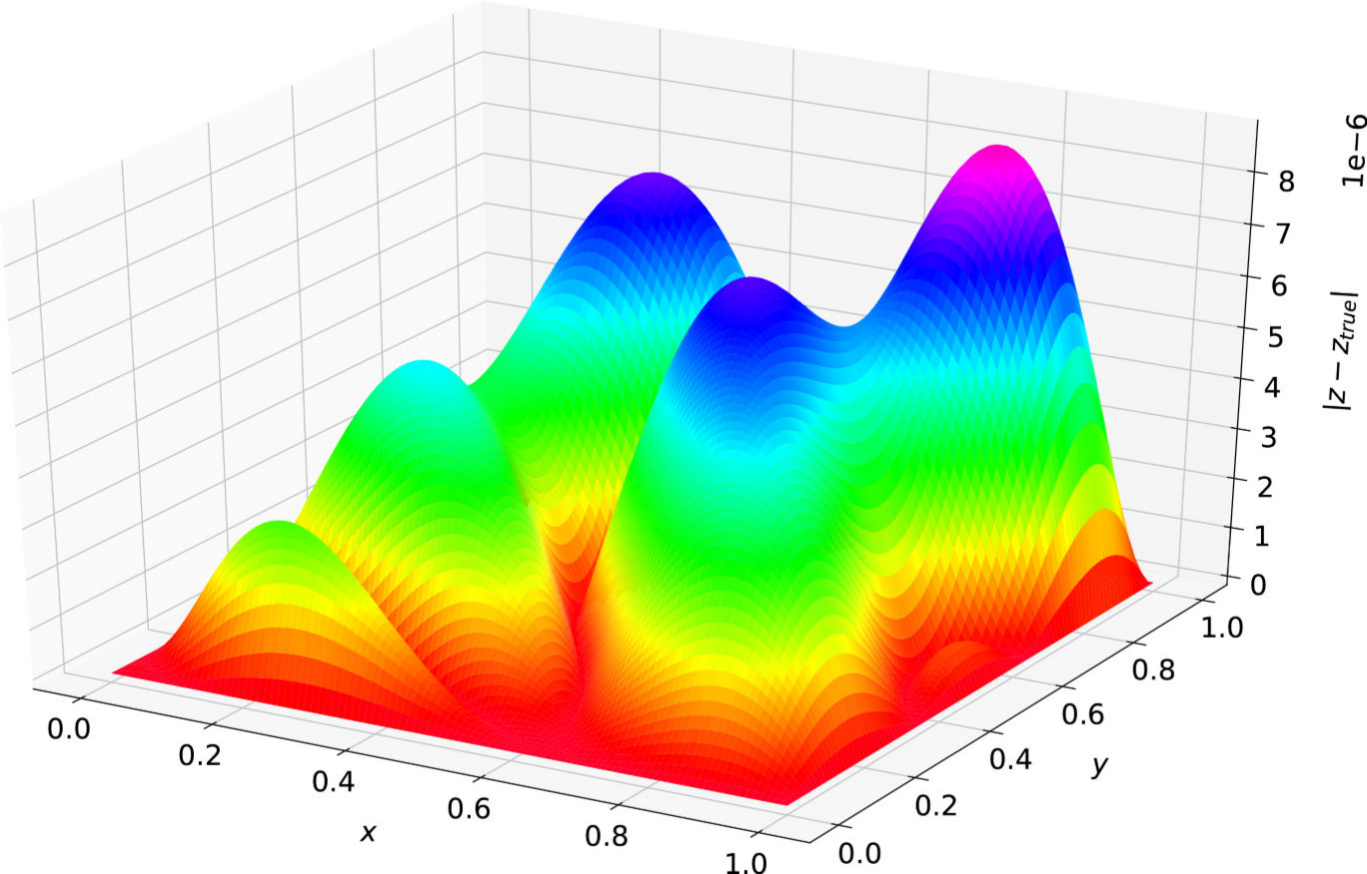
Problem 1 solution error using Deep TFC solution form.

Figure 3 was created using the solution form posed in [5]. The maximum error on the test set was 4.246 × 10^−7^and the average error on the test set was 1.133 × 10^−7^. Figure 4 was created using the Deep TFC solution form. The maximum error on the test set was 8.790 × 10^−6^ and the average error on the test set was 2.797 × 10^−6^.

Comparing Figures 3 and 4 shows that the solution form from [5] gives an estimated solution that is approximately an order of magnitude lower in terms of average error and maximum error for this problem. Hence, the more complex TFC solution form requires a more complex neural network to achieve the same accuracy as the simpler solution form from Ref. [5] with a simple neural network. This results in a trade-off. The TFC constrained expressions allow for more complex boundary conditions (i.e., derivatives of arbitrary order) and can be used on *n*-dimensional domains, but require a more complex neural network. In contrast, the simpler solution form from Ref. [5] can achieve the same level of accuracy with a simpler neural network, but cannot be used for problems with higher order derivatives or *n*-dimensional domains.

### 4.2. Problem 2

The second problem analyzed was the wave equation, shown in Equation (11).

(11) $$\frac{\partial^{2}u}{\partial t^{2}}(x,t)=c^{2}\frac{\partial^{2}u}{\partial x^{2}}(x,t)\;\text{subject}\;\text{to:}\;\left\{ \begin{array}{l} z(0,t)=0\\ z(1,t)=0\\ z(x,0)=x(1-x)\\ z_{t}(x,0)=0 \end{array}\right.\;\text{where}\;(x,y)\in [0,1]\times [0,1]$$

where the constant, *c* = 1. The analytical solution for this problem is,

$$z(x,t)=\sum_{k=0}^{\infty }\frac{8}{{\left(2k+1\right)}^{3}\pi^{3}}\sin((2k+1)\pi x)\cos((2k+1)c\pi t).$$

Although the true analytical solution is an infinite series, for the purposes of making numerical comparisons, one can simply truncate this infinite series such that the error incurred by truncation falls below machine level precision. The constrained expression for this problem is shown in Equation (12).

(12) $$f(x,t;\theta )=(1-x)[N(0,0;\theta )-N(0,t;\theta )]+x[N(1,0;\theta )-N(1,t;\theta )]-N(x,0;\theta )\;\;+x(1-x)+N(x,t;\theta )+t\left[\left(1-x)N_{t}(0,0;\theta )+xN_{t}(1,0;\theta )-N_{t}(x,0;\theta \right)\right]$$

The neural network used to estimate the solution to this PDE was a fully connected neural network with three hidden layers and 30 neurons per layer. The non-linear activation function used was the hyperbolic tangent. The biases and weights were initialized using the same method as problem 1. The training points, (*x*, *t*), were created by choosing *x* to be an independent and identically distributed (IID) random variable with uniform distribution in the range [0, 1], and *t* to be an IID random variable with uniform distribution in the range [0, 1]. The network was trained using the BFGS method and 1000 training points.

**Figure 5.**
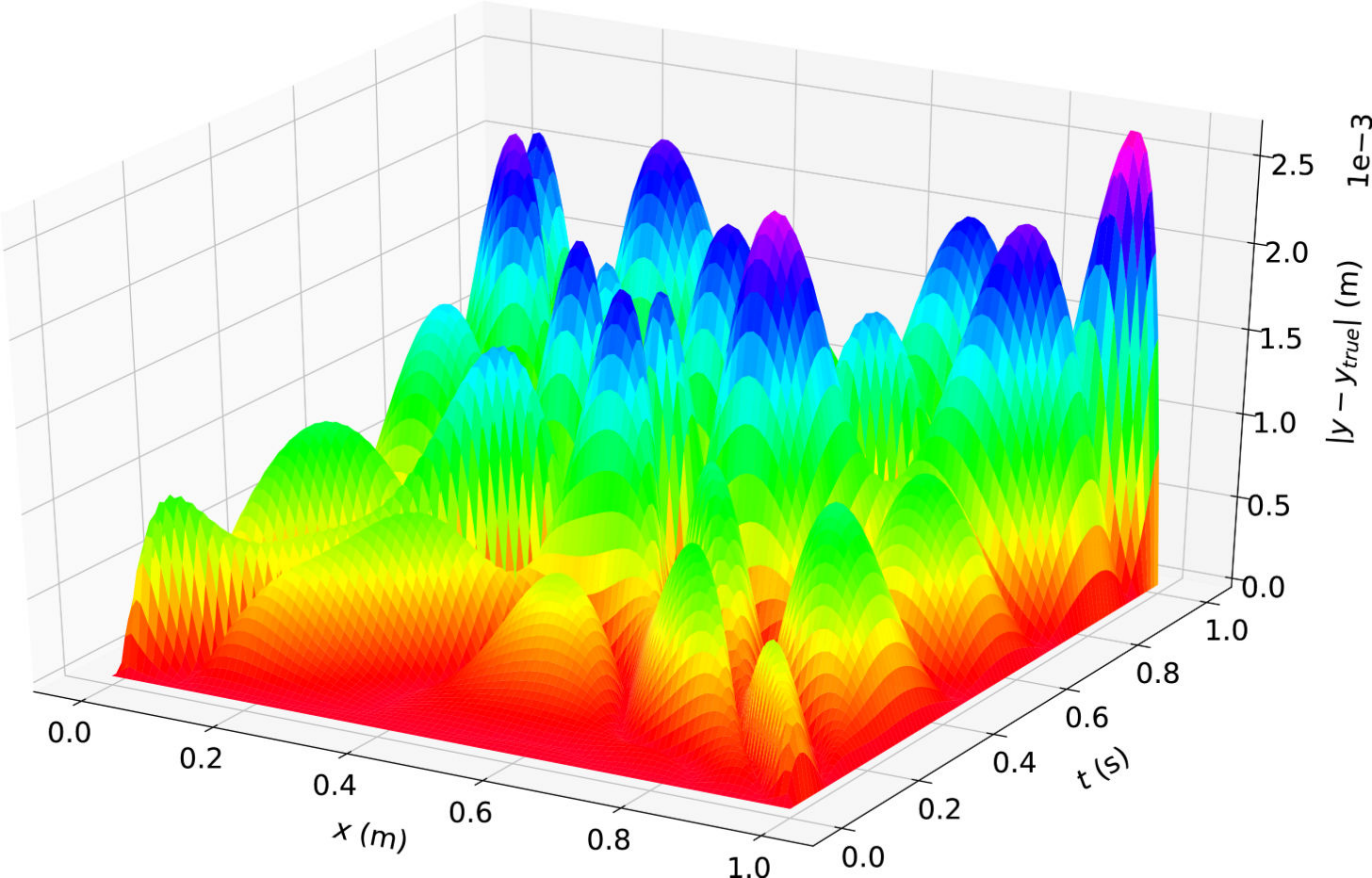
Problem 2 solution error.

Figure 5 shows the difference between the analytical solution and the estimated solution using Deep TFC on a grid of 10,000 evenly distributed points; this grid represents the test set.

The maximum error on the test set was 2.643 × 10^−3^ m and the average error on the test set was 6.305 × 10^−4^ m. The error of this solution is larger than in the problem 1, while the solution values are on the same order of magnitude, 10^−1^ m, as in problem 1. The larger relative error in problem 2 is due to the more oscillatory nature of the solution (i.e., the surface of the true solution in problem 2 is more complex than that of problem 1).

### 4.3. Problem 3

The third problem analyzed was a known solution to the incompressible Navier–Stokes equations, called Poiseuille flow. The problem solves the flow velocity in a two-dimensional pipe in steady-state with a constant pressure gradient applied in the longitudinal axis. Equation (13) shows the associated equations and boundary conditions.

(13) $$\frac{\partial u}{\partial x}+\frac{\partial v}{\partial y}=0\;\rho \left(\frac{\partial u}{\partial t}+u\frac{\partial u}{\partial x}+v\frac{\partial u}{\partial y}\right)=-\frac{\partial P}{\partial x}+\mu \left(\frac{\partial^{2}u}{\partial x^{2}}+\frac{\partial^{2}u}{\partial y^{2}}\right)\;\rho \left(\frac{\partial v}{\partial t}+u\frac{\partial v}{\partial x}+v\frac{\partial v}{\partial y}\right)=\mu \left(\frac{\partial^{2}v}{\partial x^{2}}+\frac{\partial^{2}v}{\partial y^{2}}\right)\;\text{subject}\;\text{to:}\;\left\{ \begin{array}{l} u(0,y,t)=u(L,y,t)=u(x,y,0)=\frac{1}{2\mu }\frac{\partial P}{\partial x}\left(y^{2}-{\left(\frac{H}{2}\right)}^{2}\right)\\ u\left(x,\frac{H}{2},t\right)=u\left(x,-\frac{H}{2},t\right)=0\\ v(0,y,t)=v(L,y,t)=v(x,y,0)=0\\ v\left(0,\frac{H}{2},t\right)=v\left(0,-\frac{H}{2},t\right)=0 \end{array}\right.$$

where *u* and *v* are velocities in the *x* and *y* directions respectively, *H* is the height of the channel, *P* is the pressure, *ρ* is the density, and *μ* is the viscosity. For this problem, the values *H* = 1 m, *ρ* = 1 kg/m^3^, *μ* = 1 Pa·s, and $\frac{\partial P}{\partial x}=-5\;\text{N}/{\text{m}}^{3}$ were chosen. The constrained expressions for the *u*-velocity, *f*^*u*^(*x*, *y*, *t*; _*θ*_), and *v*-velocity, *f*^*v*^(*x*, *y*, *t*; _*θ*_), are shown in Equation (14).

(14) $$f^{u}(x,y,t;\theta )=N(x,y,t;\theta )-N(x,y,0;\theta )+\frac{L-x}{L}(N(0,y,0;\theta )-N(0,y,t;\theta ))\;\;+\frac{x}{L}(N(L,y,0;\theta )-N(L,y,t;\theta ))+\frac{P\left(4y^{2}-H^{2}\right)}{8\mu }\;\;+\frac{1}{2HL}((2y-H)\left((L-x)N\left(0,-\frac{H}{2},0;\theta \right)+xN\left(L,-\frac{H}{2},0;\theta \right)-LN\left(x,-\frac{H}{2},0;\theta \right)\right)\;\;-(L-x)N\left(0,-\frac{H}{2},t;\theta \right)+LN\left(x,-\frac{H}{2},t;\theta \right)-xN\left(L,-\frac{H}{2},t;\theta \right))\;\;-(L-x)N\left(0,-\frac{H}{2},t;\theta \right)+LN\left(x,-\frac{H}{2},t;\theta \right)-xN\left(L,-\frac{H}{2},t;\theta \right))\;\;-(H+2y)((L-x)N\left(0,\frac{H}{2},0;\theta \right)-LN\left(x,\frac{H}{2},0;\theta \right)+xN\left(L,\frac{H}{2},0;\theta \right)\;\;-(L-x)N\left(0,\frac{H}{2},t;\theta \right)-xN\left(L,\frac{H}{2},t;\theta \right)+LN\left(x,\frac{H}{2},t;\theta \right)))\;f^{v}(x,y,t;\theta )=N(x,y,t;\theta )-N(x,y,0;\theta )+\frac{L-x}{L}(N(0,y,0;\theta )-N(0,y,t;\theta ))\;\;+\frac{x}{L}(N(L,y,0;\theta )-N(L,y,t;\theta ))+\frac{1}{2HL}((2y-H)\left((L-x)N\left(0,-\frac{H}{2},0;\theta \right)\right)\;\;+xN\left(L,-\frac{H}{2},0;\theta \right)-LN\left(x,-\frac{H}{2},0;\theta \right)-(L-x)N\left(0,-\frac{H}{2},t;\theta \right)+LN\left(x,-\frac{H}{2},t;\theta \right)\;\;-xN\left(L,-\frac{H}{2},t;\theta \right))-(H+2y)((L-x)N\left(0,\frac{H}{2},0;\theta \right)-LN\left(x,\frac{H}{2},0;\theta \right)\;\;+xN\left(L,\frac{H}{2},0;\theta \right)-(L-x)N\left(0,\frac{H}{2},t;\theta \right)-xN\left(L,\frac{H}{2},t;\theta \right)+LN\left(x,\frac{H}{2},t;\theta \right)))$$

The neural network used to estimate the solution to this PDE was a fully connected neural network with four hidden layers and 30 neurons per layer. The non-linear activation function used was the sigmoid. The biases and weights were initialized using the same method as problem 1. The training points, (*x*, *y*, *t*), were created by sampling *x*, *y*, and *t* IID from a uniform distribution that spanned the range of the associated independent variable. For *x*, the range was [0, 1]. For *y*, the range was $\left[-\frac{H}{2},\frac{H}{2}\right]$, and for *t*, the range was [0, 1]. The network was trained using the BFGS method on a batch size of 1000 training points. The loss function used was the sum of the squares of the residuals of the three PDEs in Equation (13).

The maximum error in the *u*-velocity was 3.308 × 10^−7^ m per second, the average error in the *u*-velocity was 9.998 × 10^−8^ m per second, the maximum error in the *v*-velocity was 5.575 × 10^−7^ m per second, and the average error in the *v*-velocity was 1.542 × 10^−7^ m per second. Despite the complexity, the maximum error and average error for this problem are six to seven orders of magnitude lower than the solution values. However, the constrained expression for this problem essentially encodes the solution, because the initial flow condition at time zero is the same as the flow condition throughout the spatial domain at any time. Thus, if the neural network outputs a value of zero for all inputs, the problem will be solved exactly. Although the neural network does output a very small value for all inputs, it is interesting to note that none of the layers have weights or biases that are at or near zero.

### 4.4. Problem 4

The fourth problem is another solution to the Navier–Stokes equations, and is very similar to the third. The only difference is that in this case, the fluid is not in steady state, it starts from rest. Equation (15) shows the associated equations and boundary conditions.

(15) $$\frac{\partial u}{\partial x}+\frac{\partial v}{\partial y}=0\;\rho \left(\frac{\partial u}{\partial t}+u\frac{\partial u}{\partial x}+v\frac{\partial u}{\partial y}\right)=-\frac{\partial P}{\partial x}+\mu \left(\frac{\partial^{2}u}{\partial x^{2}}+\frac{\partial^{2}u}{\partial y^{2}}\right)\;\rho \left(\frac{\partial v}{\partial t}+u\frac{\partial v}{\partial x}+v\frac{\partial v}{\partial y}\right)=\mu \left(\frac{\partial^{2}v}{\partial x^{2}}+\frac{\partial^{2}v}{\partial y^{2}}\right)\;\text{subject}\;\text{to:}\;\left\{ \begin{array}{l} u(0,y,t)=\frac{\partial u}{\partial x}(L,y,t)=u(x,y,0)=0\\ u\left(x,\frac{H}{2},t\right)=u\left(x,-\frac{H}{2},t\right)=0\\ v(0,y,t)=\frac{\partial v}{\partial x}(L,y,t)=v(x,y,0)=0\\ v\left(x,\frac{H}{2},t\right)=v\left(x,-\frac{H}{2},t\right)=0 \end{array}\right.$$

This problem was created to avoid encoding the solution to the problem into the constrained expression, as was the case in the previous problem. The constrained expressions for the *u*-velocity, *f*^*u*^(*x*, *y*, *t*; _*θ*_), and *v*-velocity, *f*^*v*^(*x*, *y*, *t*; _*θ*_), are shown in Equation (16).

(16) $$f^{u}(x,y,t;\theta )=N(x,y,t;\theta )-N(x,y,0;\theta )+N(0,y,0;\theta )-N(0,y,t;\theta )+xN_{x}(L,y,0;\theta )-xN_{x}(L,y,t;\theta )\;\;+\frac{1}{2H}((2y-H)\left(N\left(0,-\frac{H}{2},0;\theta \right)-N\left(x,-\frac{H}{2},0;\theta \right)+xN_{x}\left(L,-\frac{H}{2},0;\theta \right)-N\left(0,-\frac{H}{2},t;\theta \right)\right)\;\;+N\left(x,-\frac{H}{2},t;\theta \right)-xN_{x}\left(L,-\frac{H}{2},t;\theta \right))-(H+2y)(N\left(0,\frac{H}{2},0;\theta \right)-N\left(x,\frac{H}{2},0;\theta \right)\;\;+xN_{x}\left(L,\frac{H}{2},0;\theta \right)-N\left(0,\frac{H}{2},t;\theta \right)+N\left(x,\frac{H}{2},t;\theta \right)-xN_{x}\left(L,\frac{H}{2},t;\theta \right)))\;f^{v}(x,y,t;\theta )=N(x,y,t;\theta )-N(x,y,0;\theta )+N(0,y,0;\theta )-N(0,y,t;\theta )+xN_{x}(L,y,0;\theta )-xN_{x}(L,y,t;\theta )\;\;+\frac{1}{2H}((2y-H)(N\left(0,-\frac{H}{2},0;\theta \right)-N\left(x,-\frac{H}{2},0;\theta \right)+xN_{x}\left(L,-\frac{H}{2},0;\theta \right)-N\left(0,-\frac{H}{2},t;\theta \right)\;\;+N\left(x,-\frac{H}{2},t;\theta \right)-xN_{x}\left(L,-\frac{H}{2},t;\theta \right))-(H+2y)(N\left(0,\frac{H}{2},0;\theta \right)-N\left(x,\frac{H}{2},0;\theta \right)\;\;+xN_{x}\left(L,\frac{H}{2},0;\theta \right)-N\left(0,\frac{H}{2},t;\theta \right)+N\left(x,\frac{H}{2},t;\theta \right)-xN_{x}\left(L,\frac{H}{2},t;\theta \right)))$$

The neural network used to estimate the solution to this PDE was a fully connected neural network with four hidden layers and 30 neurons per layer. The non-linear activation function used was the hyperbolic tangent. The biases and weights were initialized using the same method as problem 1. Problem 4 used 2000 training points that were selected the same way as in problem 3, except the new ranges for the independent variables were [0, 15] for *x*, [0, 3] for *t*, and $\left[-\frac{H}{2},\frac{H}{2}\right]$ for *y*.

**Figure 6.**
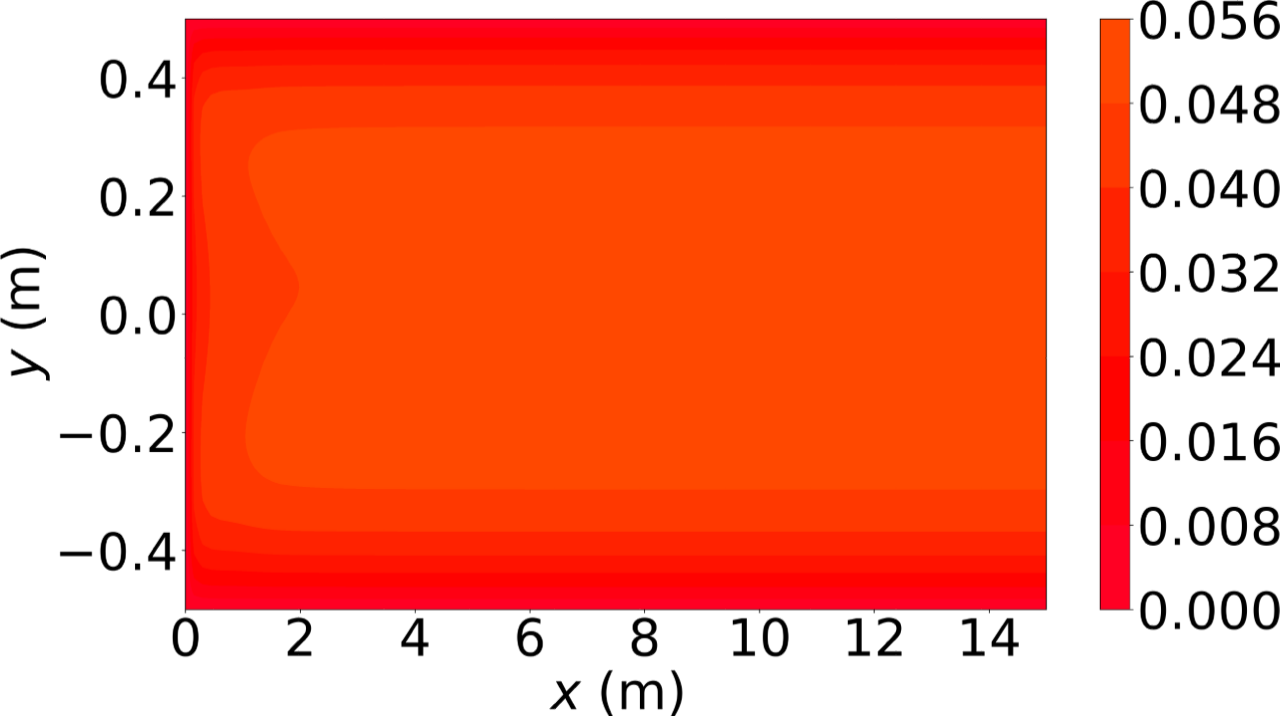
*u*-velocity in meters per second at 0.01 s.

**Figure 7.**
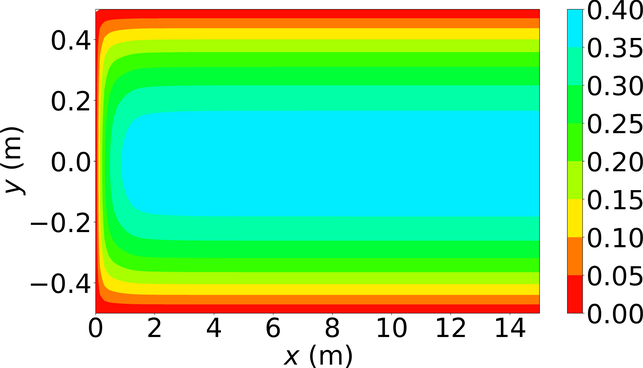
*u*-velocity in meters per second at 0.1 s.

**Figure 8.**
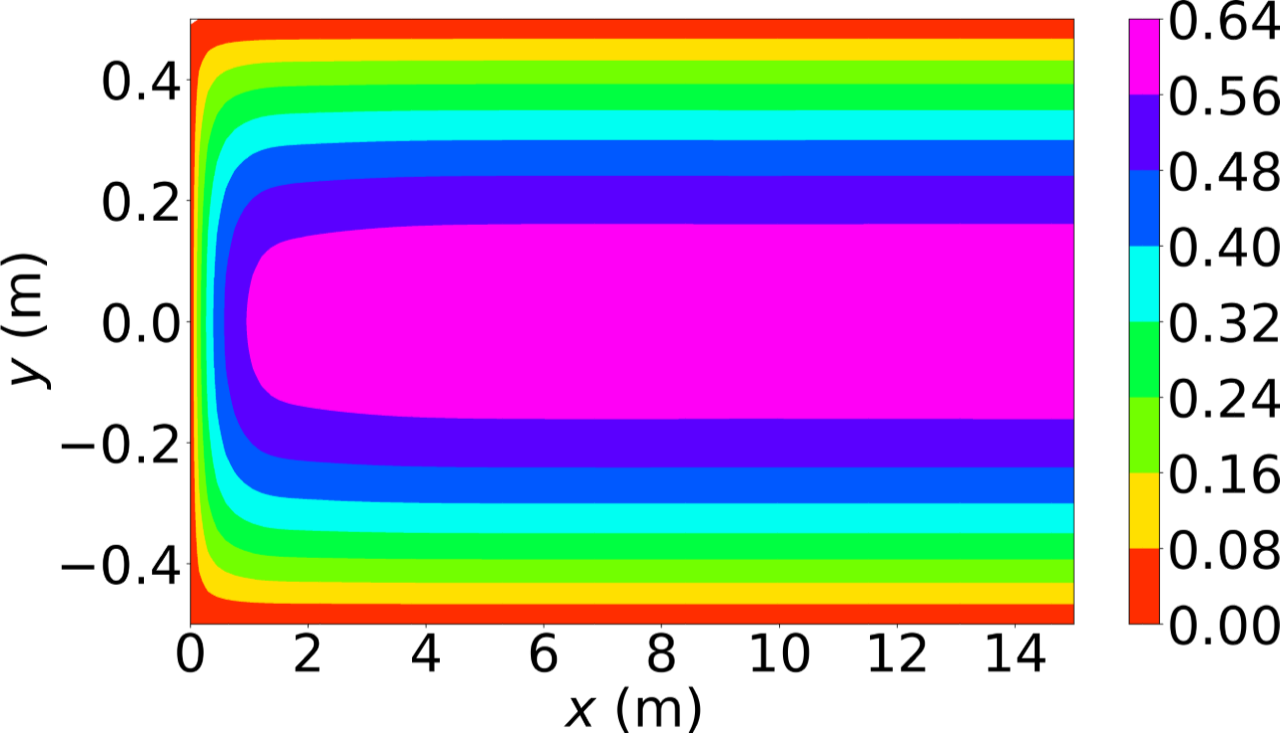
*u*-velocity in meters per second at 3.0 s.

Figures 6–8 show the *u*-velocity of the fluid throughout the domain at three different times. Qualitatively, the solution should look as follows. The solution should be symmetric about the line *y* = 0, and the solution should develop spatially and temporally such that after a sufficient amount of time has passed and sufficiently far from the inlet, *x* = 0, the *u*-velocity will be equal, or very nearly equal, to the steady state *u*-velocity of problem 3. Qualitatively, the *u*-velocity field looks correct in Figures 7 and 8, and throughout most of the spatial domain in Figure 6. However, near the left end of Figure 6, the shape of the highest velocity contour does not match that of the other figures. This stems from the fact that none of the training points fell near this location. Other numerical estimations of this PDE were made with the exact same method, but with different sets of random training points, and in those that had training points near this location, the *u*-velocity matched the qualitative expectation. However, none of those estimated solutions had a quantitative *u*-velocity with an error as low as the one shown in Figures 6–8. Quantitatively, the *u*-velocity at *x* = 15 from Figure 8 was compared with the known steady state *u*-velocity, and had a maximum error of 5.378 × 10^−4^ m per second and an average error of 3.117 × 10^−4^ m per second.

## 5. Conclusions

This article demonstrated how to combine neural networks with the Theory of Functional Connections (TFC) into a new methodology, called Deep TFC, that was used to estimate the solutions of PDEs. Results on this methodology applied to four problems were presented that display how accurately relatively simple neural networks can approximate the solutions to some well known PDEs. The difficulty of the PDEs in these problems ranged from linear, two-dimensional PDEs to coupled, non-linear, three-dimensional PDEs. Moreover, while the focus of this article was on numerically estimating the solutions of PDEs, the capability to embed constraints into neural networks has the potential to positively impact performance when solving any problem that has constraints, not just differential equations, with a neural network.

Future work should investigate the performance of different neural network architectures on the estimated solution error. For example, Ref. [4] suggests a neural network architecture where the hidden layers contain element-wise multiplications and sums of sub-layers. The sub-layers are more standard neural network layers like the fully connected layers used in the neural networks of this article. Another architecture to investigate is that of extreme learning machines [22]. This architecture is a single layer neural network where the weights of the linear output layer are the only trainable parameters. Consequently, these architectures can ultimately be trained by linear or non-linear least squares for linear or non-linear PDEs respectively.

Another topic for investigation is reducing the estimated solution error by sampling the training points based on the loss function values for the training points of the previous iteration. For example, one could create batches where half of the new batch consists of half of the points in the previous batch that had the largest loss function value and the other half are randomly sampled from the domain. This should consistently give training points that are in portions of the domain where the estimated solution is farthest from the real solution.

Finally, future work will explore extending the hybrid systems approach presented in Ref. [23] to *n*-dimensions. Doing so would enable Deep TFC to solve problems that involve discontinuities at interfaces. For example, consider a heat conduction problem that involves two slabs of different thermal conductivities in contact with one another. At the interface condition, the temperature is continuous but the derivative of temperature is not.

## Figures and Tables

**Table 1.** Comparison of Deep TFC, Ref. [5], and finite element method (FEM).

Method	Training Set	Test Set
Deep TFC	3 × 10^−7^	3 × 10^−7^
Ref. [5]	5 × 10^−7^	5 × 10^−7^
FEM	2 × 10^−8^	1.5 × 10^−5^

## Abbreviations

FEM: finite element method
IID: Independent and identically distributed
PDE: partial differential equation
TFC: Theory of Functional Connections
